# Improving Misfire Fault Diagnosis with Cascading Architectures via Acoustic Vehicle Characterization

**DOI:** 10.3390/s22207736

**Published:** 2022-10-12

**Authors:** Adam M. Terwilliger, Joshua E. Siegel

**Affiliations:** Department of Computer Science and Engineering, Michigan State University, East Lansing, MI 48824, USA

**Keywords:** acoustic recognition, cascading architecture, combustion engine, convolutional neural network, deep learning, fault diagnosis, misfire, vehicle attributes

## Abstract

In a world dependent on road-based transportation, it is essential to understand automobiles. We propose an acoustic road vehicle characterization system as an integrated approach for using sound captured by mobile devices to enhance transparency and understanding of vehicles and their condition for non-expert users. We develop and implement novel deep learning cascading architectures, which we define as conditional, multi-level networks that process raw audio to extract highly granular insights for vehicle understanding. To showcase the viability of cascading architectures, we build a multi-task convolutional neural network that predicts and cascades vehicle attributes to enhance misfire fault detection. We train and test these models on a synthesized dataset reflecting more than 40 hours of augmented audio. Through cascading fuel type, engine configuration, cylinder count and aspiration type attributes, our cascading CNN achieves 87.0% test set accuracy on misfire fault detection which demonstrates margins of 8.0% and 1.7% over naïve and parallel CNN baselines. We explore experimental studies focused on acoustic features, data augmentation, and data reliability. Finally, we conclude with a discussion of broader implications, future directions, and application areas for this work.

## 1. Introduction

Transportation is essential to daily life, with over one billion automobiles in use globally [1]. These vehicles produce approximately three billion metric tons of carbon dioxide annually [2] and consume scarce natural resources. As miles travelled increase, the need to reduce emissions and conserve energy grows in importance.

Advances in autonomy and shared mobility will also soon demand that costly highly automated vehicles be amortized over increased miles travelled [3,4,5]. A growing market for personal automobiles, alongside increased longevity requirements, decreased supply, and demand for long-life vehicles such as those to be used in new mobility services has led to more demanding needs for vehicle longevity than ever before. In turn, there is a need to maintain peak performance over longer vehicle lifetimes, as persisted inefficiencies drive the total cost of ownership higher and increase environmental damage unnecessarily.

Core to the problem of persisted inefficiency is a lack of consumer knowledge. Though there have been efforts to make waste and emissions management exciting and accessible [6] to average individuals, the same is less true for fields such as automotive maintenance. Without detailed knowledge of vehicles or their maintenance needs, automobiles are often left to languish, driven with check engine lights on and unburnt fuel exiting their exhaust pipes. There is an opportunity to build smarter vehicle diagnostics that help untrained individuals assess, evaluate, and plan a response to vehicle operating conditions.

While vehicle technology is rapidly advancing, fault diagnostics are lesser-explored, whether for trained individuals—such as mechanics, or unskilled individuals—such as vehicle owners and operators. There is a largely unmet need for a better way to understand the state of vehicles such that operators might better access the information necessary to identify and plan response to impending or latent issues. At the same time, prior work shows that bringing expert knowledge to non-experts can have significant implications for fuel and energy savings [7,8,9,10] as well as safety [11].

Sound is an efficient, easy-to-use, and cost-effective means of capturing informative mechanical data [12], e.g., by using audio captured with a smartphone to identify fault or wear states [8,9]. Compared with images [11], video, and other high-volume information, sound, like acceleration [7,10], is densely informative and compact. As a result, sound can be efficiently processed in near real-time by low-cost hardware such as embedded devices [13]. Audio is particularly useful for providing insight into systems with periodic acoustic emissions, such as the rotating assemblies commonly found in vehicles and other heavy and industrial equipment [14,15,16,17,18,19,20].

Vehicles’ internal combustion engines emit distinct and varied sound during operation. During the driving process, the engine could experience issues with its supply of fuel, intake air, and/or ignition spark. These failures could occur for reasons such as a clogged air filter or damaged ignition coil. In this manuscript, we explore the fault of an engine misfires, which occur when the volume of one cylinder fails to combust due to a lack of air, fuel, or spark. This type of fault can not only result in a drop in fuel efficiency and additional maintenance costs but also can increase long-term costs through the need for secondary repairs, and can even be dangerous if left unattended.

While a trained mechanic may be able to recognize an engine misfire by ear, it is unlikely the same could be said for the average consumer. We propose an automated system that leverages deep learning [21,22] to detect misfires across all types of engines. Our approach seeks to not only predict misfire fault diagnosis but also predict the type of the engine through multi-task learning. This is motivated through the real-world process of vehicle fault diagnosis where a mechanic would want to know the make, model, year, mileage, etc. to make a better informed decision. Our system is inspired in the same way where we first characterize the engine across four different attributes: fuel type (gasoline or Diesel), engine configuration (flat, inline, V), cylinder count (4, 6, 8), and engine aspiration (turbo-charged or normally aspirated). With these predicted attributes, our system then “cascades” these predictions as features to better inform the second stage misfire fault detection.

**Figure 1:**
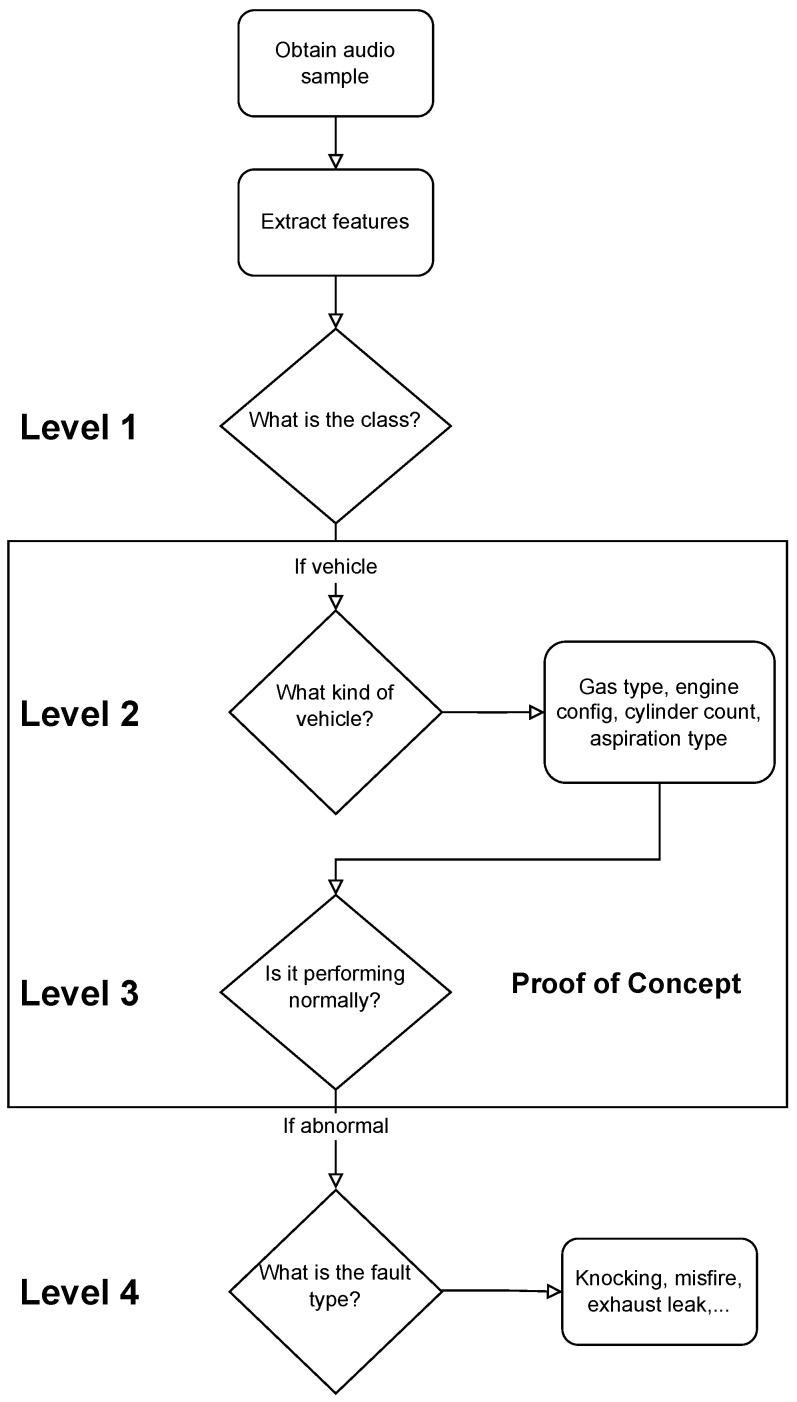
Flowchart of our proposed cascading architecture for vehicle understanding using audio data. We can see there are four sequential levels with a conditional dependence between levels 1→2 and 3→4. Of particular note is the transition between level 2 and 3, as level 2 cascades its attribute predictions to level 3. This work focuses on level 2 and 3 as a proof-of-concept.

We propose novel cascading architectures for vehicle understanding, as visualized in Figure 1. We define a cascading architecture as a multi-level, sequential, conditional network that makes multiple predictions and cascades each prediction to every successive layer of the network. We also compare this architecture to a non-cascading, parallel architecture. Our representative cascading network has four distinct layers:

1. **General acoustic classification:** Does the audio sample contain a vehicle?
2. **Attribute recognition:** What is the kind of vehicle?
3. **Status prediction:** Is the vehicle performing normally?
4. **Fault identification:** If abnormal, what fault is occurring?

In this manuscript, we identify three main areas of novelty with our work:

- **Misfire fault diagnosis**: While most misfire fault diagnostic approaches are limited to specific types of engines, we built a more robust system that can detect misfires across unique fuel types, engine configurations, cylinder counts, and engine aspirations.
- **Acoustic Vehicle Characterization**: We trained a two-stage convolutional neural network (CNN) that takes as input acoustic features and then predicts attributes of the engine. To the best of our knowledge, we built the first acoustic recognition system that can simultaneously characterize engines and diagnose faults.
- **Cascading Architectures**: We proposed a broad framework for cascading architectures and then showcased a proof-of-concept approach which achieved 87.0% test set accuracy on misfire fault detection improving 8.0% and 1.7% over naïve and non-cascading baselines.

To demonstrate the impact of our approach, we conducted ablation studies on data augmentation and model generalizability. In the conclusion, we consider the broader implications, future directions, and potential applications of our work.

## 2. Prior Art

The presented work is novel in three main areas. To establish how our work relates to and builds upon these areas, we now explore relevant prior art for each in Section 2.1, Section 2.2 and Section 2.3. First, we consider fault detection in automotive combustion engines. Second we delve into vehicle classification tasks using audio features. Finally, we compare our proposed cascading architecture to similar sequential and conditional architectures.

### 2.1. Misfire Fault Diagnosis

There is a long history of works focused on misfire fault detection in internal combustion engines. Many approaches utilize vibration analysis [23,24,25,26,27,28,29] and physics-based estimations such as with acceleration, torque, and speed [30,31,32,33].

Our work focuses on utilizing only an unrefined acoustic signal to detect the misfire. This approach has been narrowly studied, specificially with traditional fully connected artificial neural networks (ANNs) combined with chaos analysis utilizing wavelet features have been leveraged on audio collected from smartphones [34,35]. Authors [28] build ANN models that compare vibration and sound modes with Fast Fourier Transform (FFT) features. Using sound quality metrics with a support vector machine (SVM) [36,37] also improves upon vibration analysis. Parallel tasks in misfire fault analysis consider finding the location of the misfire [38] or extracting the fault component from the acoustic signal [39].

One research study looking to identify specific faults [9] collects nearly 1k audio samples of vehicles using smartphone microphones for which approximately 1/3 are abnormal “vehicle misfire” events. Using traditional machine learning techniques and various audio features combined with feature selection, [9] achieves 1.0% misclassification rate and 1.6% false positive rate. However, this previous work was trained on three vehicles types and it is unclear if and how the results would generalize to a broader sample including more diverse vehicles.

Most approaches for acoustic misfire fault diagnosis only consider a single type of engine such as gasoline [30], Diesel [24,27], turbocharged [32], or four cylinder [28,36]. The strength and novelty of our approach is that it is trained on over 200 unique engine samples that vary across fuel type, engine configuration, cylinder count, and aspiration type. So our approach is not specific to Diesel or turbocharged for example, but rather is agnostic to the engine type when detecting misfires. This makes our approach more dependable when deployed in a real-world scenario for a consumer checking the status of their vehicle.

### 2.2. Acoustic Vehicle Characterization

We define general acoustic classification [40] as classifying a signal across many labels within broad categories. AudioSet [41], for example, is a popular dataset for general acoustic classification, which contains 527 diverse, hand-annotated classes for 2.1 million YouTube videos. AudioSet [41] provides an ontology for general acoustic classification with seven categories including: human sounds (speech/body), animals, music, sounds of things (vehicle, engine, tools), natural (weather), source-ambiguous, and background noise.

Related prior art focuses on high-level audio classification using neural networks [42,43,44,45,46,47,48,49]. These works often utilize audio embeddings [50,51,52,53] with transfer learning to better inform the overall acoustic classification task. Within Audioset [41] ontology, there are numerous applications that utilize DL for sound-based tasks range from music genre classification [54,55] to medical diagnoses [56,57,58] to animal recognition [59,60,61,62,63,64] and more [65,66,67]. We build on similar classification techniques and differentiate our work in three ways: model pre-training, data source, and application.

First, we focus on multiple low-level, fine-grained multi-level label prediction with the assumption of the highest-level class, rather than simply classifying a single level at a time. Given our approach focuses on these fine-grained tasks, we do not utilize pre-trained acoustic embeddings [50,52,53] but rather train lightweight models from scratch.

Second, we utilize data collected at a higher sampling rate (48 kHz) compared to that collected from YouTube, which we show, in Section 5.2, caps its sampling rate and in the process may discard useful informative features. This is important because models trained on such crowd-sourced audio may not perform as well as models trained and operated using raw, full-frequency audio directly collected from a mobile device. YouTube and other such sources also conduct feature-destructive compression on some audio samples, which can limit model performance and generalizability.

Third, our work is novel in its application of sound-based deep learning, as we focus on acoustic vehicle characterization. Prior art [12] has conducted an extensive survey on off-board vehicle vibro-acoustic diagnostics which explores a multitude of acceleration- and sound-based vehicle characterization approaches. Our work focuses on the lesser-explored tasks of vehicle attribute recognition, engine misfire fault detection, and specifically the interrelation between vehicle variant and fault-specific diagnostic algorithms.

Previous work has focused on vehicle attribute recognition [12,68] utilizing traditional ML techniques such as SVMs, random forests, and feature selection. These primarily consider the FFT and Mel-Frequency Cepstral Coefficients (MFCC) feature types, whereas our work considers five distinct feature types and conducts an in-depth feature comparison. These works also do not consider the engine configuration attribute task. Our work explores the three previously explored attribute prediction tasks: fuel type, cylinder count, aspiration type, as well as the engine configuration task. Refs. [12,68] also build separate models for each prediction task, whereas our deep learning approach demonstrates that predicting attributes jointly in a single model can yield strong results, with variant-identifying parameters helping to “shape” the final diagnostic model.

Our work is unique in its merging of vehicle acoustic characterization tasks. First, classifying attributes for an abnormally performing vehicle may be a more challenging task than has been explored [12,68] for normally performing vehicles. Second, we show the value of multi-task learning through attributes prediction improving misfire performance and vice versa. Finally, we demonstrate a proof-of-concept with our two-stage CNN that both characterizes engines and diagnoses faults.

### 2.3. Cascading Architectures

We envision our cascading architecture for vehicle understanding as a sequential, conditional model. Specifically, we construct an architecture with four sequential levels (Figure 1), where levels 1→2 and 3→4 are conditionally dependent. Additionally, level 3 is dependent upon the cascaded result from level 2. There is valuable related work in sequential and conditional deep learning that has been utilized in a select applications. Examples include human activity recognition [69], image segmentation [70], facial recognition [71], frame prediction in games [72], and music rhythm generation [73]. Three works are of particular relevance to our cascading architecture for acoustic vehicle characterization: [74,75,76].

The approach from [74] has elements that are similar to our proposed cascading architecture. Specifically, [74] leverages the relationship between fine and coarse labels in their model training. The first and second level of our cascading architecture can be seen as examples of coarse and fine labels, which have a subset relationship. However, [74] does not consider conditional logic and does not have multiple levels like our proposed cascading architecture.

Both [75] and our work utilize neural networks for acoustic classification. However, the conditional component of [75] does not correspond with multi-label dependence proposed in our cascading architecture but rather conditional dependence in the data itself. Specifically, [75] leverages the temporal relationship of nearby frames in spectrograms whereas our work utilizes the entire spectrogram, agnostic to its temporal nature, as a feature set in the CNN.

CascadeML [76] is closely related to our proposed architecture. Our architecture might almost be considered an example of the theoretic structure proposed by [76]. However, ref. [76] focuses on the AutoML component where the network can dynamically grow itself based upon any number of multi-label samples. We instead embed expert knowledge into the static structure of the cascading architecture. In other words, we know fault type is conditional upon status prediction, so we embed that in the structure of our architecture rather than building a network from scratch with the AutoML approach of [76]. Not only will this approach allow us to demonstrate stronger and more consistent performance, but also it would be theoretically more efficient given it structure is already defined.

In summary, refs. [74,75,76] showcase sequential, conditional, and cascading architectures that are closely related to our work. However, these works focus on either theoretical or high level acoustic classification. We show there is value in building a model for the specific application of acoustic vehicle characterization and misfire fault diagnosis.

## 3. Approach

As noted in the problem motivation, proper maintenance is increasingly important to vehicles as their number and miles travelled grow. However, as vehicles become more like appliances and less familiar to their users, driver and passenger awareness of their capabilities diminishes. We suggest that automated vehicle identification is an important first step in diagnosing and rectifying problems plaguing efficiency and emissions: when a part fails, it is not enough to know that a vehicle is big or small, or red or silver, but rather that a vehicle is a 2016 version of some specific make and model possessing a particular engine and transmission type and tire size. Creating tools that effectively identify vehicles and use these attributes as a means of improving state characterization allows non-expert users to expertly diagnose their own vehicles, clearing the knowledge hurdle to effective maintenance and thereby reducing fuel consumption, emissions, and likelihood of breakdown.

Our approach is a proof-of-concept for how vehicle characteristics can better inform fault diagnosis and we explain our approach from five main angles: Section 3.1, Section 3.2, Section 3.3, Section 3.4 and Section 3.5. First, we share the details of our novel dataset, specifying the dataset splits and augmentation procedure. Second, we compare the different audio feature types used in our models. Third, we develop a high-level understanding of the two multi-task models proposed in this work. Then, we clarify the specifics of the CNNs for each main feature type. Finally, we provide implementation details to aid in reproducibility.

### 3.1. Dataset

Our dataset is a collection of audio samples recorded using mobile phones. We utilize mobile phone microphones as these tend to have repeatable characteristics within the range of frequency similar to those of human speech and hearing (roughly 20 Hz–20 kHz). In particular, we merge the datasets from [12,68], which focus on attributes, with [9,77], which focus on misfire detection. We outline the makeup of our dataset and its splits in Table 1.

Together, these datasets totaled 286 (228+58) audio samples, which contributed to about 320 min of non-augmented audio. We decided to split each audio sample into three second clips (“chunks”) to provide our features and models with a standard input size for training and to expand our dataset size. This time was selected as striking an appropriate balance of capturing low-frequency sounds with ease and speed of capture. Additionally, we believe three seconds is a long enough sample to be representative data for our model, but also short enough that would allow for clips within the same sample to be distinct and useful for training. After chunking the samples, this resulted in 6406 (5455+951) unique three second clips. Though fixed-length clips were used, our models at test time are agnostic to the input feature size, rather than needing the sample to be exactly three seconds.

Our approach for the train/validation/test split involved building a train set using only augmented clips with the goal of reducing overfitting and improving generalizability. In turn, we split the real full length samples into a validation and test set, using a traditional 80/20 split. As seen in Table 1, this resulted in 5455 validation clips and 951 test clips. We split the validation and test set using the raw samples rather then the clips because we created the train set using only the validation set. This allowed for the test set to be mutually exclusive from both the train and validation set to showcase as accurate as possible the model’s outsample generalizability.

**Figure 2:**
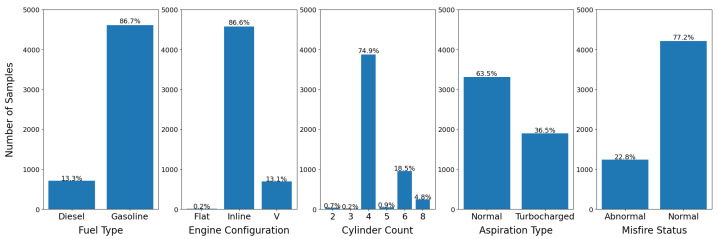
Label distribution for the validation set. We observe the classes are not perfectly balanced and some labels in the engine configuration and cylinder count are very underrepresented.

**Figure 3:**
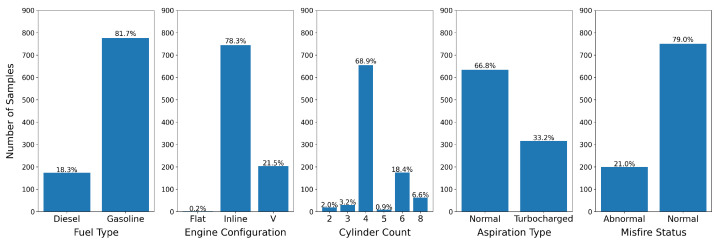
Label distribution for the test set. We attempted to build a test set with as similar label distribution to training and validation as possible, while also including at least one raw sample from each label.

Due to the relatively small size of the datasets, we manually created the validation and test splits with an attempt to balance the label distribution to the best of our ability for the four attribute recognition tasks: fuel type, engine configuration, cylinder count, and aspiration type and the status prediction task focused on misfire detection. Label distributions are visualized in Figure 2 and Figure 3.

#### 3.1.1. Data Augmentation

In deep learning for computer vision, data augmentation is often utilized via rotations, cropping, scaling, etc. to gain invariance among unique inputs. Audio may similarly be augmented. For instance, ref. [78] utilized time stretching, dynamic range compression, pitch shifting, and background noise for environmental sound classification. In speech recognition, ref. [79] applied multiple speed changes to expand their training set. Focused on animal audio classification, ref. [80] uniquely applies computer vision augmentation techniques to the spectrogram such as reflection and rotation, as well as waveform augmentation with pitch, speed, volume, random noise, and time shift. For music classification, ref. [81] found pitch-shifting to be most informative but also utilizes time stretching and random frequency filtering.

Researchers [82] classify accelerating vehicles using audio and apply random noise and pitch shift to expand their dataset. Other work [83] focuses on vehicle type identification using MFCCs and other audio features, these authors apply city noise to augment captured signals. There have been additional applications of augmentation to acoustic vehicle DL related tasks [84,85,86]. Our work is similar to [78,80,81] in that we utilize pitch, speed, volume, and random noise augmentation types. However, we differentiate ourselves in our application of the augmentation to multi-task learning for vehicle attribute recognition and fault detection. Additionally, to our knowledge, our approach is unique in that our train set is not a combination of real and augmented samples but is entirely made up of augmented samples. We hypothesize this may allow for more robust training and less overfitting / memorizing of specific samples.

Our process of creating the training set consisted of applying a data augmentation process eight times to each of the 5455 validation clips, which when expanded resulted in a train set of size 43,640 as seen in Table 1. The data augmentation process consisted of applying four types of data augmentation to each clips which each data augmentation type having a probability of 50% of being activated. The types of augmentation chosen were volume change, pitch shift, speed shift, and random background noise. More specifically, we chose each type of augmentation from a randomly uniformly distribution over these augmentation parameters:

- Change volume between −5.0 and 5.0.
- Pitch shift between −0.25 and 0.25.
- Change speed between 0.92 and 1.08.
- Add background noise with signal to noise ratio (SNR) between 0.05 and 0.20.

These ranges were set based on performing at limit, listening, and determining sample was recognizable with each applied individually. We also added an epsilon between $10^{-6}$ and $10^{-5}$ to each parameter, so as to not randomly choose a hyperparameter of zero volume change/pitch shift or one for speed change, which would result in the clip not being augmented at all. Additionally, if the clip was not changed after all four augmentation types rolled their probability (i.e., all four types were not activated), we applied one augmentation type at random. This guaranteed that no training clips were exactly the same as their respective validation clip. All value ranges were determined experimentally, with expert mechanic review to coarsely validate the “closeness” of the augmented sample with the original sample. Augmentation parameters were set so as to line up with typical variation in engine configurations, e.g., through manufacturing diversity and wear. For example, frequency shift was bounded based on typical allowable tolerances for idle speed. Amplitude limits were set so as to minimize the effect of signal clipping. The features were determined to be acoustically discernible after augmentation.

In summary, we created a new dataset with a non-traditional train/validation/test split. This set uses only augmented variants of validation samples in training and is mutually exclusive from both the validation and test set. Our dataset, per Table 1, in total consisted of 50,046 three second clips for a total of 2502.3 minutes or about 42 h total of audio. We further motivate our decision for using data augmentation later in Section 5.1.

### 3.2. Features

**Figure 4:**
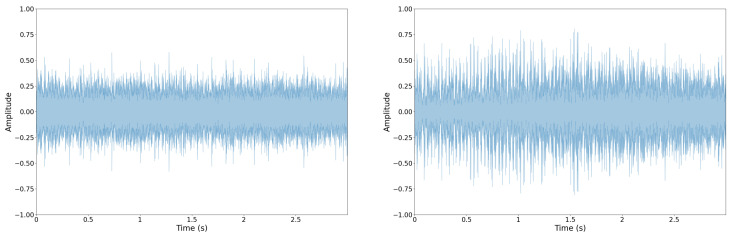
Example of two acoustic vehicle samples as raw waveforms: normal (**left**) and abnormal/misfire (**right**). Using the waveform to differentiate between vehicle types is a challenging task.

**Figure 5:**
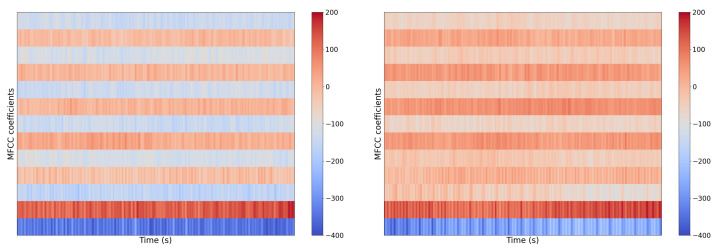
Example of two acoustic vehicle samples transformed as Mel-Frequency Cepstral Coefficients (MFCCs): normal (**left**) and abnormal/misfire (**right**). Compared to the raw waveform from Figure 4, the MFCCs provide more informative features for fault diagnosis.

The raw audio signal, known as a waveform, can provide standalone insights, but often feature extraction and transformation is a necessary step in building successful models in some acoustic recognition tasks [87,88]. Transforming the raw waveform from the time domain to the frequency domain with the provides particularly informative features. Other informative feature types utilize hybrid time and frequency information. Figure 4 and Figure 5 show a representative waveform and Mel-Frequency Cepstral Coefficients (MFCCs) for two automotive engine types; note how the MFCCs are more visually discernable. This characteristic better informs our CNN models.

In this work, we explore five audio feature types which include:

- Fast Fourier Transform (FFT)
- Mel-Frequency Cepstral Coefficients (MFCCs)
- Spectrogram
- Waveform
- Wavelets

These features were chosen to give our models a diverse set of inputs. The raw waveform provides the model with time information, while the FFT provides the model with frequency information. MFCCs, spectrograms, and wavelets provide the model with varying degrees of hybrid time and frequency information at different dimensionality. MFCCs and spectrograms are 2D features while FFT, waveform, and wavelets are 1D. Distinct models are trained for each feature type.

### 3.3. Model

We design and implement a variant of the earlier-proposed Cascade model: a two-stage network for which the first stage specializes on attributes classification and the second stage specializes on misfire detection. The first stage receives one of the previously described feature sets as input. The second stage receives both the feature set and the attribute predictions from the first stage. Both stages are trained jointly using multi-task learning.

To compare against the Cascade model, we also consider two baselines: Naïve and Parallel models. The Naïve model is a straightforward baseline that predicts the most represented class for each task. For example, if we look at Figure 3, the Naïve model would always predict gasoline as the fuel type. This model would achieve 81.7% test accuracy.

**Figure 6:**
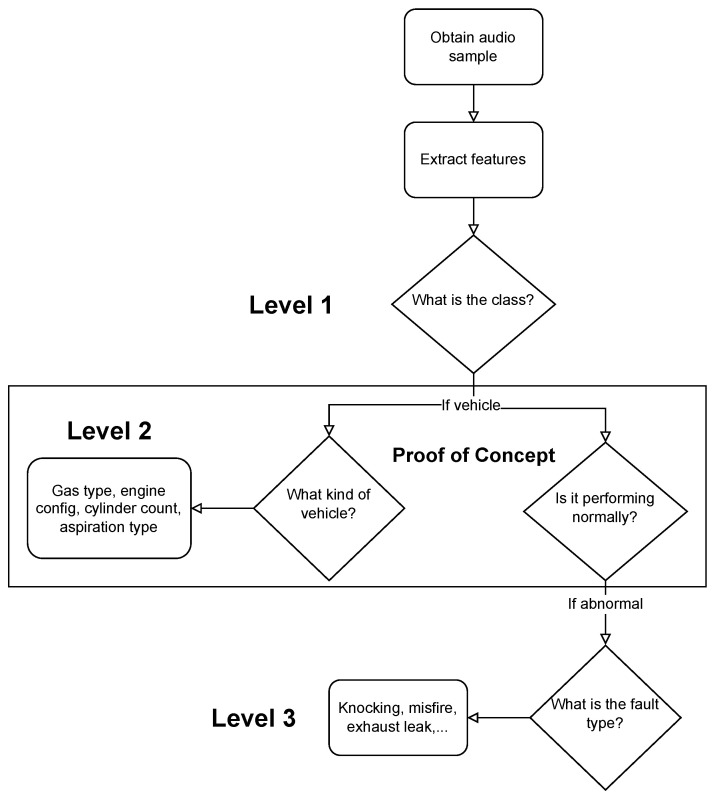
Flowchart of our baseline Parallel model architecture. The Parallel model is differentiated from the Cascade model in that rather than level 2 cascading to level 3, we combine these levels in parallel.

The Parallel model, as seen in Figure 6, represents a more complex baseline to consider than the Naïve model. The Parallel model is represented as a sequential, multi-level, conditional network in the same archetype as the Cascade model. However, per Figure 6, it does not explicitly cascade, or provide as input, the classified vehicle type to the status prediction level. Rather, these two tasks are predicted in parallel from the raw waveform and/or features.

**Figure 7:**
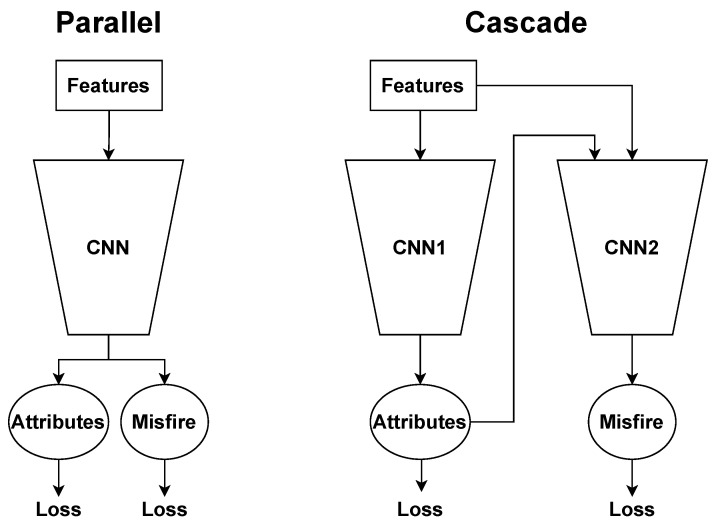
Parallel and Cascade CNN model architecture comparison. We observe the Parallel model uses a shared representation for both the attributes and misfire predictions whereas the Cascade model has two distinct CNNs that allow for specialization in attributes and misfire. Additionally, both networks utilize multi-task learning so the attributes loss can inform the misfire loss and vice versa.

Both the Cascade and Parallel model are viable classification approaches: both serve as a proof-of-concept for a full scale AI mechanic architecture and provide an all-in-one solution for classifying attributes and misfire. The Cascade and Parallel models also both utilize multi-task learning to allow for the attributes prediction to better inform the misfire prediction and vice versa. However, the models differ in their internal sharing of intermediate classification representations. Therefore, there is value in the comparison on whether a joint, shared representation via the Parallel model or separate, specialized representations via the Cascade model would yield better performance. It is also worth noting that given the Cascade model has a second sequential stage, there is an non-negligible increase in model runtime and memory. In turn, these factors should be worth considering if the Cascade model provides a margin on any task predictions. These two models are directly compared in Figure 7.

### 3.4. Network

We propose two distinct multi-layer convolutional neural networks (CNNs), Parallel and Cascade, to predict both vehicle attributes and engine misfire, as seen in Figure 7.

For each, we group audio features into 1D (FFT, waveform, wavelets) and 2D (Spectrogram, MFCCs) sets, with two two distinct model architectures that utilize 1D and 2D convolution, respectively, motivated by [89]. We adapt the M5 architecture [90] which was built for general audio classification using the raw waveform.

Our samples utilize a 48 kHz sampling rate, for which we consider frequencies ≤24 kHz according to the Nyquist–Shannon Sampling Theorem. These samples were collected using a stereo microphone for which we average the dual channel input into a single mono channel. We split each sample into 3 s chunks which results in a 1 × 72,000 input vector for the raw waveform.

The unique aspect of the M5 model utilizes a large convolutional kernel size of 80 in the first layer to propagate a large receptive field through the network. We adopt kernel size of 3 from [90] for the remaining three convolutional layers.

For the MFCC model, we utilize kernel size of 2×2 since we chose 13 MFCC coefficients as a hyperparameter which results in an input dimensionality of 130×13. For the spectrogram, we choose hyperparameters of 512 for hop length and 2048 for window size. This results in a input dimensionality of 1025×282. Since it was a larger input dimension than the MFCCs, we utilize traditional 3×3 kernels for spectrogram.

For the 2D models, our model consists of three convolution layers, rather than four layers for the 1D models because of their smaller input width. We follow the precedent set by [90] for including pooling, batchnorm, and ReLU after each conv layer in both 1D and 2D models. We also add dropout with probability =0.5 after each layer to improve generalizability and to minimize the likelihood of overfitting. We treat each prediction task as classification and therefore have final fully connected output layers with dimensionality corresponding to the number of classes for each task. These predictions are then fed into log softmax and trained using negative log-likelihood loss. Specifically, we consider five loss terms which are all equally weighted:

$$L_{\mathrm{total}}=\frac{1}{w}\left(L_{\mathrm{fuel}}+L_{\mathrm{config}}+L_{\mathrm{cyl}}+L_{\mathrm{turbo}}+L_{\mathrm{misfire}}\right)$$

### 3.5. Implementation

We built our models using PyTorch within an Anaconda environment. We trained our models using 1080 Ti and Titan XP GPUs. Our models unless otherwise stated were trained for 100 epochs with the Adam optimizer, utilizing a batch size of 128, learning rate of 0.001, no weight decay, and with amsgrad following the standard set by [90] for training CNNs on audio. The Spectrogram model did not fit into GPU memory with a batch size of 128, so a batch size of 64 and 32 were used for the Parallel and Cascade models, respectively.

We conducted a handful of hyperparameter searches to find the best set for each model type, across number of epochs, learning rate, and dropout rate. Specifically, we looked at number of epochs ranging from 5 to 100, learning rate ranging from 0.0001 to 0.1, and dropout rate ranging from 0.0 to 0.75. These experiments did not yield any notable trend and in turn have not included these results in our manuscript. Results exploring validation performance, confusion matrices, and hyperparameters can be found in [91]. However, any model weights and log files are available via request for reproduciblility. For the following Section 4 and Section 5, we utilize the hyperparameters which result in the top performing model for each feature type.

Our dataset and code are undergoing an IP review and are expected to be released to the public.

## 4. Results

We evaluate classifier performance along two dimensions in Section 4.1 and Section 4.2. For model comparison, we first compare our Parallel and Cascade architectures as seen in Figure 7. Our goal is to provide unbiased data and to learn the scenarios under which each model performs better or worse than the other. We hypothesize that the Cascade model will demonstrate better performance on the misfire fault detection task over the Parallel approach. Next, with the feature comparison subsection, we illustrate the impact the choice of audio feature has on model accuracy. Finally, we delve into task comparison to better understand how our top performing models are classifying each of the four main attributes: fuel type, engine configuration, cylinder count, and aspiration type, as well these labels’ contribution to misfire fault detection performance. We include evaluation metrics (precision/recall) and confusion matrices to provide additional insight into the true value of our approaches.

### 4.1. Model Comparison

Table 2 compares the test set accuracy for each feature type between the Naïve, Parallel and Cascade models. With respect to the Naïve baseline, it appears that the Parallel and Cascade models plateau for attributes. However, for misfire prediction the Cascade model outperforms both the Naïve and Parallel baselines across every feature type. Specifically, when comparing Parallel and Cascade model test set accuracy on the misfire task, we observe 4.0%, 1.5%, 1.0%, 8.8%, 1.7% improvements for FFT, MFCC, spectrogram, waveform, and wavelet model performance respectively.

**The major takeaway: the Cascade model outperforms all baselines on misfire prediction. These findings show we have achieved our goal of improving fault prediction through cascading of vehicle attributes.**

### 4.2. Feature Comparison

We explore model performance for five feature types (FFT, MFCC, spectrogram, wavelets, waveform). FFT, wavelets, and waveform share the exact same model architecture, whereas MFCC and spectrogram each use a unique model architecture with differing amounts of layers and kernel sizes to reflect the feature dimensionality.

When exploring the test set performance from Table 3, we find MFCC is the top performing feature for the Parallel and Cascade models. We also note that the models with spectrogram features achieve second place for both Parallel and Cascade on the test set. This shows that the models which utilize 2D convolution are able to find more generalizable patterns from the train set to the test set. The final pattern we can observe is the poor performance of the raw waveform relative to the other feature types. This demonstrates the value of our experiments which analyze many different types of audio features.

## 5. Ablation Studies

To prove model robustness for previously unseen vehicles and vehicle variants, we conducted two ablation studies in Section 5.1 and Section 5.2. As discussed in Section 3.1.1, data augmentation is an essential component of modern deep learning systems. We first explore how our models perform on the test set when trained with and without data augmentation. Secondly, we built an additional test set to consider our models outsample generalizability by using input data crowd-sourced from YouTube. Through these test set experiments, we demonstrate why this is not an effective approach to evaluating real-world model performance. In particular, training on crowdsourced data from YouTube is not well-suited to model creation that is broadly generalizable for acoustic vehicle characterization tasks.

### 5.1. Data Augmentation

Our first ablation study explores the difference of training on augmented samples vs. real samples on model performance. Table 4 compares across each of the five feature types for test set accuracy. We observe a strong improvement on misfire prediction accuracy when trained on augmented samples for all models. More specifically, we find 6.4%, 4.6%, 3.7%, 4.2%, and 5.7% margins of improvement when using data augmentation for misfire detection with the FFT, MFCC, spectrogram, waveform, and wavelet models, respectively.

When exploring the attributes recognition tasks, some feature type models utilize data augmentation better than others. For example, the waveform performs better across all four attributes tasks when trained on augmented samples. This makes sense since there would be more room for the network to memorize and overfit to the raw data. Additionally, the spectrogram model improves on all four tasks with augmentation, the MFCC and wavelet models improve on 3/4 tasks. The FFT model had a small degradation in attributes tasks with augmentation which is reasonable given it had the biggest margin of improvement for misfire prediction.

In summary, data augmentation is a crucial component of training effective acoustic classification networks. We showed with our Cascade model that training on augmented samples has a positive impact across all feature types for misfire fault detection.

### 5.2. YouTube Outsample

We collected 51 samples from YouTube (YT) to provide our models with representative data for evaluating outsample generalizability. After chunking these samples every three seconds, we built a new test set of 649 never-before-seen clips to evaluate the outsample performance of our model.

We compare the test set performance of our Cascade models to the YT outsample performance in Table 5. We observe a significant performance degradation of our models on these YouTube clips. Specifically, we find across all feature types, there is minimal to extreme degradation in all five prediction tasks. With the top-performing MFCC model, we see 5.2%, 36.5%, 54.4%, 1.2%, and 22.6% decreases in accuracy across the fuel type, engine configuration, cylinder count, aspiration type, and misfire status tasks, respectively.

**Figure 8:**
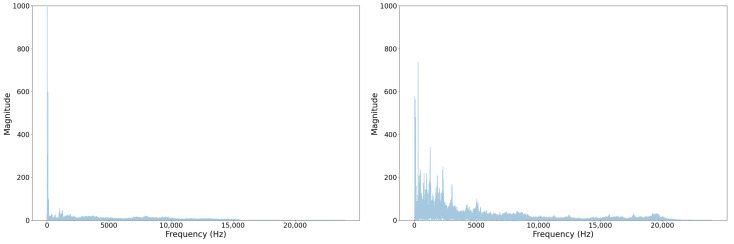
Example FFT—YT Sample—Frequency attenuated (**left**) and regular sample (**right**).

**Figure 9:**
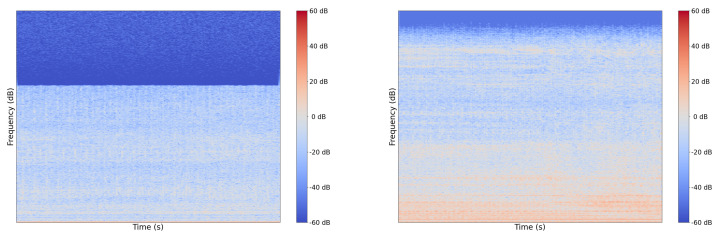
Example Spectrogram—YT Sample—Frequency attenuated (**left**) and regular sample (**right**).

There are a handful of reasons why this sort of discrepancy has occurred between our test set and the YouTube test set. It could have resulted from a lack of sufficient data and a label distribution mismatch compared to the training set. However, we have evidence that YouTube audio is frequency-attenuated on certain videos, likely to save space. We show an example of a regular sample from our set and a sample from the YouTube set in Figure 8 and Figure 9. In Figure 8, note that there is no representation of frequencies greater than 16 kHz in the YT sample whereas in raw device-captured samples we see frequencies represented well beyond 20 kHz. Given our sampling rate is 48 kHz, according to the Nyquist–Shannon Theorem, our features should have informative frequency representation up to 24 kHz.

From a physics standpoint, some features, such as turbocharger bearing rotation, would make acoustic emissions around these higher frequencies. The comparison of Spectrograms shown in Figure 9 further elucidates the frequency attentuation. For the Youtube sample, in Figure 9, we can visualize a sharp knee in the function at a much lower frequency than appears in the regular sample Spectrogram.

In summary, we showed that there should be caution applied when working with crowd-sourced acoustic data such as that in datasets originating on YouTube. This further strengthens the need for more comprehensive data collection and generation to create a balanced and diverse set for testing the outsample performance of models.

## 6. Discussion

We will explore three main areas of discussion in Section 6.1, Section 6.2 and Section 6.3. Within broader implications, we consider the larger impact our proposed architecture may have on both consumers and industry. For future directions, we note routes this work could be continued and built upon with respect to data quality, model improvement, and architectural modifications. Finally, we discuss the how our work could be utilized in diverse application areas within and outside vehicle characterization and diagnostics.

### 6.1. Broader Implications

There are two main groups to consider for the broader impacts of our work developing an AI mechanic: consumers and industry.

Given the end-to-end nature of our proposed system, it has practical usability for consumers to obtain vehicle status in real-time, e.g., using a mobile phone. Our high-level cascading architecture takes as input a raw audio sample, requiring no direct input or knowledge from the consumer. This has the impact of potentially saving even non-expert users stress, time, and money. Whenever their vehicle may be exhibiting a fault, they can receive an initial opinion from the AI mechanic which could provide the consumer with insight into the seriousness of their vehicle state.

There are multiple extensions for the AI mechanic when consumer data are integrated. A feedback loop would be created that could improve the system for detecting faults. The collection of fault data over time from long-standing consumers would create the opportunity for preventive maintenance. One day, the AI mechanic may help average consumer to prevent their vehicle from exhibiting a fault in the first place through proper warnings.

Prognostics will not come about immediately. For there to be value in a feedback loop from consumers, vehicles must be allowed to fail such that these data might be captured. Additionally, we showed the impact underrepresented labels have on our model and its poor outsample performance on most underrepresented labels. Without a more diverse, comprehensive, and well-balanced dataset, this behavior will still be observed. When the AI mechanic is working with a previously unseen vehicle, it will take the system time to adjust, and may necessitate the use of techniques such as synthetic data generation or federated learning.

Within the automotive industry, off-board diagnostics which can utilize inexpensive microphones and mobile or embedded devices an alternative to the costly expense of on-board diagnostics that constantly collect and send data from the vehicle. The AI mechanic could provide automakers with an enormous swath of data on the status condition and fault identification for each kind of vehicle.

Our cascading models are flexible in that despite being trained using the vehicle attribute predictions as input to the second stage network, this could easily be adapted to take the ground truth labels as input instead. The value of this change could be significant for automakers as it would allow them to build unique fault identification models conditional upon specific vehicle types. If automakers could use inexpensive audio data to create a chart of anticipated vehicle wear, this may be used to design better vehicles and supporting infrastructure. This will require the capture and labeling of samples for desired fault identification types. If other fault types were to be integrated into the AI mechanic, this would require hundreds or thousands of distinct examples across vehicle classes to achieve similar performance to our method’s demonstrated misfire detection performance.

### 6.2. Future Directions

Throughout our results and ablation sections, we brought up issues with our current approach and raised potential opportunities for improvement. One of the main issues uncovered (and not unique to the AI mechanic) is the models’ dependency on quality data that is not only diverse but also balanced across classes. We showed that obtaining crowd-sourced audio data from YouTube may address data quantity, but not data quality. There is value in developing a real-world application that would make it easy for a consumer to collect data on their mobile device and store the waveform at its original 48 kHz sampling rate to prevent lossy compression or frequency attenuation.

Another avenue for future work to address the lack of data, especially in underrepresented classes, would be generative modeling such as with Generative Adversarial Networks (GANs) [92] and Diffusion Models [93]. Generative models could be extended not only to acoustic samples, but also utilized in a feedback loop for developing better synthetic samples for training, including for classes not well represented. One direction for improving test set accuracy would be exploring feature and model fusion. Considering an ensemble approach where 1D and 2D convolution can be utilized on their respective feature types should yield better overall performance. Additionally, we may consider data balancing [94] and mixup [95,96] data augmentation as ways to improve results.

There is a significant opportunity in the space of acoustic vehicle characterization. Our dataset had labels for make, OEM, idling status, recording environment/location, and more. Perhaps there could be value derived from more expansive attribute classification. Additionally, our set contains labels for horsepower and engine displacement, which would provide an interesting exploration in attribute regression.

Finally, building upon our initial proof-of-concept for a cascading architecture would be a challenging but worthwhile endeavor. It could be tackled in two main ways. The first way would be to connect more stages to the architecture both in the high-level, general acoustic classification, and low-level, fault type recognition. These additional stages may benefit from utilizing pre-trained audio embeddings and transfer learning [50,51,52,53]. The other approach, motivated by work discussed in Section 2.3, would be to implement conditional logic into the network itself. Since fault type recognition is conditional upon there being a fault in the first place, having a network which is adaptive would be a major contribution. There has already been great strides made in deep sequential networks [97] and adaptive networks focused on efficiency [98,99,100]. As such, building this work upon prior art would be a natural extension going forward.

### 6.3. Potential Applications

While our main focus of this work was on vehicle understanding, our approach and proposed cascading architecture can be adapted in broader application areas. These application areas include sequential, conditional, or multi-label prediction tasks and fault identification.

Our proposed cascading architecture is a multi-level network where each level is conditional upon the prior. Any application area where there is potential for inter-label dependency could leverage the cascading architecture. For example, music recognition has hierarchies and label dependency such as first asking does the sample contain music. Then the next stage is conditional upon the first stage where it could look to predict genre, artist, song, etc. Another area for cascading architecture could be animal recognition. Does the sample first contain an animal, then what kind of animal, what state is the animal in, what location is the animal in, is the animal behaving normally, etc.?

Not only can the cascading architecture be extended to other audio applications, but also applications with other modes of data such as in computer vision. For example, biometrics could first ascertain whether a sample contains a valid fingerprint, iris, or facial scan. Then conditionally upon the first level, it could then ask whether the biometrics scan represents a valid user, what condition the user is in, perhaps using multi-modal data such as heart rate or blood pressure prediction.

The other significant application area for these techniques is broader fault identification, particularly using audio data. This includes other diagnostic areas, such as for industrial processes or energy sector equipment. One such example is home or industrial heating and ventilation systems. Other applications include home appliances (washer/dryer) with belt slipping or drum imbalance, electric cars/bicycles with suspension issues, manufacturing equipment (CNC mills/lathes) with tool run-out or spindle issues, drills with brush wear or belt slip, the energy sector with turbine and pump health, elevator/escalators condition, and even carnival/fair equipment.

In summary, our proposed method is broadly applicable to any task which can leverage a conditional dependency and/or has a potential need for fault identification. We plan to explore the identified areas and more in continuations of this work. We believe this work can one day be extended as a real-world mobile application that can help people better understand and diagnose issues with their vehicles.

## 7. Conclusions

In this manuscript, we identified three main areas of novelty with our work:

- **Misfire fault diagnosis**: An engine misfire is a common fault that occurs in automotive vehicles that can lower fuel efficiency and increase maintenance costs. We proposed an automated deep learning system that leverages the cost-effective and prevalent audio data to diagnose misfires. Most acoustic misfire detection approaches consider only a single or small number of vehicles which limits the type of engine able to be reliably diagnosed by the resulting algorithms. Our approach is built on over 200 unique samples varied across gasoline and Diesel, I4, V6, V8, turbocharged, and more. In turn, we built a more robust fault diagnostic system.
- **Acoustic Vehicle Characterization**: Often the first step in diagnosing an issue with an automotive vehicle is to identify its characteristics such as make, model, year, mileage, etc. In our work, we look to identify four distinct attributes of the engine: fuel type, engine configuration, cylinder count, and aspiration type. Prior work has characterized these attributes using traditional machine learning techniques on normally performing samples. We merged two datasets to enable attribute recognition for the less studied abnormal misfiring samples. Additionally, our approach improved upon prior work with multi-task learning to predict four attributes and misfire in a single model.
- **Cascading Architectures**: We defined cascading architectures as sequential, multi-level, conditional networks. As a proof-of-concept, we built a two-stage convolutional neural network where the first stage specialized on vehicle attributes and then cascaded its attribute predictions to the second stage which specialized on misfire detection with the goal of better informing both prediction tasks. Our cascading model achieved 87.0% test set accuracy on misfire fault detection which demonstrates margins of 8.0% and 1.7% over naïve and non-cascading baselines.

We further explored the impact of our approach through relevant ablation studies on data augmentation and model generalizability. We concluded our manuscript with an important discussion of the broader implications, future directions, and potential applications, highlighting the potential for future research and adoption of the developed architectures and concepts.

## Figures and Tables

**Table 1:** Summary of the train/validation/test split for our datasets. We create 8 augmented training samples per one validation sample. The test sets is mutually exclusive from the train and validation sets, which allows it to provide an insight into the outsample performance and model generalizability.

Dataset Split	Raw Samples	Clips (3 s)	Total Length (min)
Train	–	43,640	2182.0
Validation	228	5455	272.75
Test	58	951	47.55
Total	286	50,046	2502.3

**Table 2:** Model comparison for test set accuracy. We observe relatively similar performance across attributes between Parallel and Cascade, while the Cascade model consistently improves upon the Parallel model for misfire prediction. Top performing model (Parallel vs. Cascade) in **bold**.

Model	Feature	Fuel	Config	Cyl	Turbo	Misfire
Naïve	–	81.7%	78.3%	68.9%	66.8%	79.0%
Parallel	FFT	78.8%	**78.3%**	**67.7%**	**70.6%**	79.5%
Cascade	FFT	**81.7%**	77.8%	68.1%	66.5%	**83.5%**
Parallel	MFCC	**84.7%**	78.3%	72.0%	**73.0%**	85.3%
Cascade	MFCC	81.7%	**79.0%**	**72.8%**	69.2%	**87.0%**
Parallel	Spectrogram	77.0%	**78.3%**	**68.9%**	65.9%	84.9%
Cascade	Spectrogram	**81.7%**	**78.3%**	**68.9%**	**67.2%**	**85.9%**
Parallel	Waveform	73.3%	75.2%	**64.7%**	**67.7%**	70.8%
Cascade	Waveform	**82.0%**	**78.3%**	52.5%	67.3%	**79.6%**
Parallel	Wavelets	**79.9%**	**63.4%**	52.7%	**69.0%**	81.4%
Cascade	Wavelets	78.4%	62.1%	**53.1%**	66.8%	**83.1%**

**Table 3:** Feature comparison for test set accuracy. For the Parallel model, MFCC features translate their strong validation performance to test set generalizability. However, the Cascade model better utilizes FFT and spectrogram features for attributes and spectrogram and wavelet features for misfire. Top performing feature type for each model in **bold**.

Model	Feature	Fuel	Config	Cyl	Turbo	Misfire
Parallel	MFCC	**84.7%**	**78.3%**	**72.0%**	**73.0%**	**85.3%**
Parallel	Spectrogram	77.0%	**78.3%**	68.9%	65.9%	84.9%
Parallel	Wavelets	79.9%	63.4%	52.7%	69.0%	81.4%
Parallel	FFT	78.8%	**78.3%**	67.7%	70.6%	79.5%
Parallel	Waveform	73.3%	75.2%	64.7%	67.7%	70.8%
Cascade	MFCC	81.7%	**79.0%**	**72.8%**	**69.2%**	**87.0%**
Cascade	Spectrogram	81.7%	78.3%	68.9%	67.2%	85.9%
Cascade	FFT	81.7%	77.8%	68.1%	66.5%	83.5%
Cascade	Wavelets	78.4%	62.1%	53.1%	66.8%	83.1%
Cascade	Waveform	**82.0%**	78.3%	52.5%	67.3%	79.6%

**Table 4:** Data augmentation test accuracy results with the Cascade model. Top performing model (with or without augmentation) in **bold**.

Feature	Aug	Fuel	Config	Cyl	Turbo	Misfire
FFT	N	81.6%	**78.2%**	**68.8%**	**67.1%**	77.1%
FFT	Y	**81.7%**	77.8%	68.1%	66.5%	**83.5%**
MFCC	N	80.0%	75.4%	66.1%	**72.5%**	82.4%
MFCC	Y	**81.7%**	**79.0%**	**72.8%**	69.2%	**87.0%**
Spectrogram	N	80.9%	**78.4%**	**68.9%**	66.7%	82.2%
Spectrogram	Y	**81.7%**	78.3%	**68.9%**	**67.2%**	**85.9%**
Waveform	N	76.1%	66.6%	49.5%	63.2%	75.4%
Waveform	Y	**82.0%**	**78.3%**	**52.5%**	**67.3%**	**79.6%**
Wavelets	N	**81.7%**	29.3%	25.3%	**68.5%**	77.4%
Wavelets	Y	78.4%	**62.1%**	**53.1%**	66.8%	**83.1%**

**Table 5:** YouTube outsample compared to traditional test set performance for the Cascade model. We find across all feature types and prediction tasks either a significant degradation or a marginal improvement when comparing the YouTube outsample vs. traditional test set.

Feature	Set	Fuel	Config	Cyl	Turbo	Misfire
FFT	Test	81.7%	77.8%	68.1%	66.5%	83.5%
FFT	YT	77.0%	35.2%	11.2%	45.7%	67.3%
MFCC	Test	81.7%	79.0%	72.8%	69.2%	87.0%
MFCC	YT	76.5%	42.5%	18.4%	68.0%	64.4%
Spectrogram	Test	81.7%	78.3%	68.9%	67.2%	85.9%
Spectrogram	YT	80.6%	36.7%	11.4%	54.6%	63.2%
Waveform	Test	82.0%	78.3%	52.5%	67.3%	79.6%
Waveform	YT	71.6%	41.6%	24.1%	52.5%	62.0%
Wavelets	Test	78.4%	62.1%	53.1%	66.8%	83.1%
Wavelets	YT	72.1%	43.6%	35.2%	54.2%	63.6%

## Data Availability

Non-augmented source data for training the models in this study will be made available on the Harvard Dataverse upon clearing necessary intellectual property reviews. Please contact the authors to access a subset of these data prior to their publication.

## References

[1] Voelcker J. (2014). 1.2 Billion Vehicles on World’s Roads Now, 2 Billion by 2035: Report. https://www.greencarreports.com/news/1093560_1-2-billion-vehicles-on-worlds-roads-now-2-billion-by-2035-report.

[2] Tiseo I. (2021). Passenger Car Carbon Dioxide Emissions Worldwide 2010–2020. https://www.statista.com/statistics/1107970/carbon-dioxide-emissions-passenger-transport/.

[3] (2018). SAE International. Definitions for Terms Related to Shared Mobility and Enabling Technologies.

[4] Bimbraw K. Autonomous cars: Past, present and future a review of the developments in the last century, the present scenario and the expected future of autonomous vehicle technology. Proceedings of the 2015 12th International Conference on Informatics in Control, Automation and Robotics (ICINCO).

[5] Bagloee S.A., Tavana M., Asadi M., Oliver T. (2016). Autonomous vehicles: Challenges, opportunities, and future implications for transportation policies. J. Mod. Transp..

[6] Papamichael I., Pappas G., Siegel J.E., Zorpas A.A. (2022). Unified waste metrics: A gamified tool in next-generation strategic planning. Sci. Total. Environ..

[7] Siegel J.E., Bhattacharyya R., Sarma S., Deshpande A. (2015). Smartphone-based wheel imbalance detection. Proceedings of the Dynamic Systems and Control Conference.

[8] Siegel J.E., Bhattacharyya R., Kumar S., Sarma S.E. (2017). Air filter particulate loading detection using smartphone audio and optimized ensemble classification. Eng. Appl. Artif. Intell..

[9] Siegel J., Kumar S., Ehrenberg I., Sarma S., Berendt B., Bringmann B., Fromont É., Garriga G., Miettinen P., Tatti N., Tresp V. (2016). Engine Misfire Detection with Pervasive Mobile Audio. Proceedings of the Machine Learning and Knowledge Discovery in Databases.

[10] Siegel J., Bhattacharyya R., Sarma S., Deshpande A., Bi Y., Kapoor S., Bhatia R. (2018). Smartphone-Based Vehicular Tire Pressure and Condition Monitoring. Proceedings of the SAI Intelligent Systems Conference (IntelliSys) 2016.

[11] Siegel J.E., Sun Y., Sarma S., Aiello M., Yang Y., Zou Y., Zhang L.J. (2018). Automotive Diagnostics as a Service: An Artificially Intelligent Mobile Application for Tire Condition Assessment. Proceedings of the Artificial Intelligence and Mobile Services—AIMS 2018.

[12] Siegel J., Coda U., Terwilliger A. (2021). Surveying Off-Board and Extra-Vehicular Monitoring and Progress Towards Pervasive Diagnostics. arXiv.

[13] Siegel J.E., Pratt S., Sun Y., Sarma S.E. (2018). Real-time Deep Neural Networks for internet-enabled arc-fault detection. Eng. Appl. Artif. Intell..

[14] Henriquez P., Alonso J.B., Ferrer M.A., Travieso C.M. (2013). Review of automatic fault diagnosis systems using audio and vibration signals. IEEE Trans. Syst. Man Cybern. Syst..

[15] Henríquez P., Alonso J., Ferrer M., Travieso C., Gómez G. (2012). Fault diagnosis using audio and vibration signals in a circulating pump. Journal of Physics: Conference Series.

[16] Yang M., Zhou W., Song T. (2020). Audio-based fault diagnosis for belt conveyor rollers. Neurocomputing.

[17] Peng C., Li Z., Yang M., Fei M., Wang Y. (2020). An audio-based intelligent fault diagnosis method for belt conveyor rollers in sand carrier. Control Eng. Pract..

[18] Liu W., Chen Z., Zheng M. An audio-based fault diagnosis method for quadrotors using convolutional neural network and transfer learning. Proceedings of the 2020 American Control Conference (ACC).

[19] Mohammed T.S., Rasheed M., Al-Ani M., Al-Shayea Q., Alnaimi F. (2020). Fault diagnosis of rotating machine based on audio signal recognition system: An efficient approach. Int. J. Simul. Syst. Sci. Technol..

[20] Jiang X.p., Cao G.q. Belt conveyor roller fault audio detection based on the wavelet neural network. Proceedings of the 2015 11th International Conference on Natural Computation (ICNC).

[21] LeCun Y., Bengio Y., Hinton G. (2015). Deep learning. Nature.

[22] Goodfellow I., Bengio Y., Courville A. (2016). Deep Learning.

[23] Cavina N., Corti E., Minelli G., Serra G. (2002). Misfire Detection Based on Engine Speed Time-Frequency Analysis. SAE Trans..

[24] Li S., Zhang Y., Wang L., Xue J., Jin J., Yu D. A CEEMD method for diesel engine misfire fault diagnosis based on vibration signals. Proceedings of the 2020 39th Chinese Control Conference (CCC).

[25] Jafarian K., Mobin M., Jafari-Marandi R., Rabiei E. (2018). Misfire and valve clearance faults detection in the combustion engines based on a multi-sensor vibration signal monitoring. Measurement.

[26] Du C., Jiang F., Ding K., Li F., Yu F. (2021). Research on Feature Extraction Method of Engine Misfire Fault Based on Signal Sparse Decomposition. Shock Vib..

[27] Qin C., Jin Y., Tao J., Xiao D., Yu H., Liu C., Shi G., Lei J., Liu C. (2021). DTCNNMI: A deep twin convolutional neural networks with multi-domain inputs for strongly noisy diesel engine misfire detection. Measurement.

[28] Firmino J.L., Neto J.M., Oliveira A.G., Silva J.C., Mishina K.V., Rodrigues M.C. (2021). Misfire detection of an internal combustion engine based on vibration and acoustic analysis. J. Braz. Soc. Mech. Sci. Eng..

[29] Goyal D., Dhami S., Pabla B. (2021). Vibration response-based intelligent non-contact fault diagnosis of bearings. J. Nondestruct. Eval. Diagn. Progn. Eng. Syst..

[30] Zheng T., Zhang Y., Li Y., Shi L. (2019). Real-time combustion torque estimation and dynamic misfire fault diagnosis in gasoline engine. Mech. Syst. Signal Process..

[31] Liu Z., Wu K., Ding Q., Gu J.X. (2020). Engine misfire diagnosis based on the torsional vibration of the flexible coupling in a diesel generator set: Simulation and experiment. J. Vib. Eng. Technol..

[32] Liu B., Zhao C., Zhang F., Cui T., Su J. (2013). Misfire detection of a turbocharged diesel engine by using artificial neural networks. Appl. Therm. Eng..

[33] Song Q., Gao W., Zhang P., Liu J., Wei Z. (2019). Detection of engine misfire using characteristic harmonics of angular acceleration. Proc. Inst. Mech. Eng. Part D J. Automob. Eng..

[34] Lima T.L.d.V., Filho A.C.L., Belo F.A., Souto F.V., Silva T.C.B., Mishina K.V., Rodrigues M.C. (2021). Noninvasive Methods for Fault Detection and Isolation in Internal Combustion Engines Based on Chaos Analysis. Sensors.

[35] Rodrigues N.F., Brito A.V., Ramos J.G.G.S., Mishina K.D.V., Belo F.A., Lima Filho A.C. (2022). Misfire Detection in Automotive Engines Using a Smartphone through Wavelet and Chaos Analysis. Sensors.

[36] Singh S., Potala S., Mohanty A.R. (2019). An improved method of detecting engine misfire by sound quality metrics of radiated sound. Proc. Inst. Mech. Eng. Part D J. Automob. Eng..

[37] Goyal D., Dhami S., Pabla B. (2020). Non-contact fault diagnosis of bearings in machine learning environment. IEEE Sens. J..

[38] Ghazaly N.M., Abdel-Fattah M., Makrahy M.M. (2019). Determination of Engine Misfire Location using Artificial Neural Networks. Int. J. Veh. Struct. Syst. (IJVSS).

[39] Dayong N., Changle S., Yongjun G., Zengmeng Z., Jiaoyi H. (2016). Extraction of fault component from abnormal sound in diesel engines using acoustic signals. Mech. Syst. Signal Process..

[40] Hershey S., Chaudhuri S., Ellis D.P., Gemmeke J.F., Jansen A., Moore R.C., Plakal M., Platt D., Saurous R.A., Seybold B. CNN architectures for large-scale audio classification. Proceedings of the 2017 IEEE International Conference on Acoustics, Speech and Signal Processing (ICASSP).

[41] Gemmeke J.F., Ellis D.P.W., Freedman D., Jansen A., Lawrence W., Moore R.C., Plakal M., Ritter M. Audio Set: An ontology and human-labeled dataset for audio events. Proceedings of the Processing IEEE ICASSP 2017.

[42] Battaglino D., Lepauloux L., Evans N., Mougins F., Biot F. (2016). Acoustic scene classification using convolutional neural networks. arXiv.

[43] Bae S.H., Choi I., Kim N.S. Acoustic scene classification using parallel combination of LSTM and CNN. Proceedings of the Detection and Classification of Acoustic Scenes and Events 2016 Workshop (DCASE2016).

[44] McLoughlin I., Zhang H., Xie Z., Song Y., Xiao W., Phan H. (2017). Continuous robust sound event classification using time-frequency features and deep learning. PLoS ONE.

[45] Sharan R.V., Moir T.J. (2017). Robust acoustic event classification using deep neural networks. Inf. Sci..

[46] Weiping Z., Jiantao Y., Xiaotao X., Xiangtao L., Shaohu P. Acoustic scene classification using deep convolutional neural network and multiple spectrograms fusion. Proceedings of the Detection and Classification of Acoustic Scenes and Events (DCASE).

[47] Aslam M.A., Sarwar M.U., Hanif M.K., Talib R., Khalid U. (2018). Acoustic classification using deep learning. Int. J. Adv. Comput. Sci. Appl..

[48] Huang J., Lu H., Lopez Meyer P., Cordourier H., Del Hoyo Ontiveros J. Acoustic scene classification using deep learning-based ensemble averaging. Proceedings of the Detection and Classification of Acoustic Scenes and Events 2019 Workshop (DCASE2019).

[49] Abeßer J. (2020). A review of deep learning based methods for acoustic scene classification. Appl. Sci..

[50] Arandjelovic R., Zisserman A. Look, listen and learn. Proceedings of the IEEE International Conference on Computer Vision.

[51] Cramer J., Wu H.H., Salamon J., Bello J.P. Look, listen, and learn more: Design choices for deep audio embeddings. Proceedings of the ICASSP 2019-2019 IEEE International Conference on Acoustics, Speech and Signal Processing (ICASSP).

[52] Kong Q., Cao Y., Iqbal T., Wang Y., Wang W., Plumbley M.D. (2020). Panns: Large-scale pretrained audio neural networks for audio pattern recognition. IEEE/ACM Trans. Audio Speech Lang. Process..

[53] Gong Y., Chung Y.A., Glass J. (2021). Ast: Audio spectrogram transformer. arXiv.

[54] Jeong I.Y., Lee K. Learning Temporal Features Using a Deep Neural Network and its Application to Music Genre Classification. Proceedings of the ISMIR.

[55] Oramas S., Barbieri F., Nieto Caballero O., Serra X. (2018). Multimodal deep learning for music genre classification. Trans. Int. Soc. Music. Inf. Retr..

[56] Chen T.E., Yang S.I., Ho L.T., Tsai K.H., Chen Y.H., Chang Y.F., Lai Y.H., Wang S.S., Tsao Y., Wu C.C. (2016). S1 and S2 heart sound recognition using deep neural networks. IEEE Trans. Biomed. Eng..

[57] Dascalu A., David E. (2019). Skin cancer detection by deep learning and sound analysis algorithms: A prospective clinical study of an elementary dermoscope. EBioMedicine.

[58] Brown C., Chauhan J., Grammenos A., Han J., Hasthanasombat A., Spathis D., Xia T., Cicuta P., Mascolo C. (2020). Exploring automatic diagnosis of COVID-19 from crowdsourced respiratory sound data. arXiv.

[59] Mac Aodha O., Gibb R., Barlow K.E., Browning E., Firman M., Freeman R., Harder B., Kinsey L., Mead G.R., Newson S.E. (2018). Bat detective—Deep learning tools for bat acoustic signal detection. PLoS Comput. Biol..

[60] Stowell D., Wood M.D., Pamuła H., Stylianou Y., Glotin H. (2019). Automatic acoustic detection of birds through deep learning: The first Bird Audio Detection challenge. Methods Ecol. Evol..

[61] Xie J., Zhu M. (2019). Handcrafted features and late fusion with deep learning for bird sound classification. Ecol. Inform..

[62] Zhong M., Castellote M., Dodhia R., Lavista Ferres J., Keogh M., Brewer A. (2020). Beluga whale acoustic signal classification using deep learning neural network models. J. Acoust. Soc. Am..

[63] Zgank A. (2021). IoT-based bee swarm activity acoustic classification using deep neural networks. Sensors.

[64] Bravo Sanchez F.J., Hossain M.R., English N.B., Moore S.T. (2021). Bioacoustic classification of avian calls from raw sound waveforms with an open-source deep learning architecture. Sci. Rep..

[65] Liu H., Li L., Ma J. (2016). Rolling bearing fault diagnosis based on STFT-deep learning and sound signals. Shock Vib..

[66] Torres R., Battaglino D., Lepauloux L. (2017). Baby cry sound detection: A comparison of hand crafted features and deep learning approach. Proceedings of the International Conference on Engineering Applications of Neural Networks.

[67] Lee C.H., Jwo J.S., Hsieh H.Y., Lin C.S. (2020). An intelligent system for grinding wheel condition monitoring based on machining sound and deep learning. IEEE Access.

[68] Coda U. (2020). Artificial Intelligence for Vehicle Engine Classification and Vibroacoustic Diagnostics. Master’s Thesis.

[69] Yang H., Yuan C., Xing J., Hu W. SCNN: Sequential convolutional neural network for human action recognition in videos. Proceedings of the 2017 IEEE International Conference on Image Processing (ICIP).

[70] Wang Q., Yuan C., Liu Y. (2019). Learning deep conditional neural network for image segmentation. IEEE Trans. Multimed..

[71] Xiong C., Zhao X., Tang D., Jayashree K., Yan S., Kim T.K. Conditional Convolutional Neural Network for Modality-Aware Face Recognition. Proceedings of the IEEE International Conference on Computer Vision (ICCV).

[72] Wang E., Kosson A., Mu T. (2017). Deep Action Conditional Neural Network for Frame Prediction in Atari Games.

[73] Makris D., Kaliakatsos-Papakostas M., Kermanidis K.L. (2018). Deepdrum: An adaptive conditional neural network. arXiv.

[74] Sun Y., Ghaffarzadegan S. An ontology-aware framework for audio event classification. Proceedings of the ICASSP 2020-2020 IEEE International Conference on Acoustics, Speech and Signal Processing (ICASSP).

[75] Medhat F., Chesmore D., Robinson J. (2020). Masked conditional neural networks for sound classification. Appl. Soft Comput..

[76] Pakrashi A., Mac Namee B. (2019). CascadeML: An automatic neural network architecture evolution and training algorithm for multi-label classification. Proceedings of the International Conference on Innovative Techniques and Applications of Artificial Intelligence.

[77] Siegel J. (2017). Automotive Engine Air Filter Audio Samples—Free Flowing, Contaminated and Obstructed Samples.

[78] Salamon J., Bello J.P. (2017). Deep convolutional neural networks and data augmentation for environmental sound classification. IEEE Signal Process. Lett..

[79] Ko T., Peddinti V., Povey D., Khudanpur S. Audio augmentation for speech recognition. Proceedings of the Sixteenth Annual Conference of the International Speech Communication Association.

[80] Nanni L., Maguolo G., Paci M. (2020). Data augmentation approaches for improving animal audio classification. Ecol. Inform..

[81] Schlüter J., Grill T. Exploring Data Augmentation for Improved Singing Voice Detection with Neural Networks. Proceedings of the ISMIR.

[82] Yang A.C., Goodman E.D. (2019). Audio Classification of Accelerating Vehicles.

[83] Chen H., Zhang Z. (2021). Hybrid neural network based on novel audio feature for vehicle type identification. Sci. Rep..

[84] Barak O., Sallem N. (2019). Audio data augmentation for road objects classification by an Artificial Neural Network. Proceedings of the Audio Engineering Society Convention 147.

[85] Bu S.J., Moon H.J., Cho S.B. Adversarial Signal Augmentation for CNN-LSTM to Classify Impact Noise in Automobiles. Proceedings of the 2021 IEEE International Conference on Big Data and Smart Computing (BigComp).

[86] Sharif M., Hotwani M., Huseyin S., Lückemeyer G. iMobilAkou: The Role of Machine Listening to Detect Vehicle using Sound Acoustics. Proceedings of the 2021 The 5th International Conference on Advances in Artificial Intelligence (ICAAI).

[87] Geiger J.T., Schuller B., Rigoll G. Large-scale audio feature extraction and SVM for acoustic scene classification. Proceedings of the 2013 IEEE Workshop on Applications of Signal Processing to Audio and Acoustics.

[88] Sharma G., Umapathy K., Krishnan S. (2020). Trends in audio signal feature extraction methods. Appl. Acoust..

[89] Kiranyaz S., Avci O., Abdeljaber O., Ince T., Gabbouj M., Inman D.J. (2021). 1D convolutional neural networks and applications: A survey. Mech. Syst. Signal Process..

[90] Dai W., Dai C., Qu S., Li J., Das S. Very deep convolutional neural networks for raw waveforms. Proceedings of the 2017 IEEE International Conference on Acoustics, Speech and Signal Processing (ICASSP).

[91] Terwilliger A.M., Siegel J.E. (2022). The AI Mechanic: Acoustic Vehicle Characterization Neural Networks. arXiv.

[92] Goodfellow I., Pouget-Abadie J., Mirza M., Xu B., Warde-Farley D., Ozair S., Courville A., Bengio Y. (2014). Generative adversarial nets. Adv. Neural Inf. Process. Syst..

[93] Ho J., Jain A., Abbeel P. (2020). Denoising diffusion probabilistic models. Adv. Neural Inf. Process. Syst..

[94] Chen H., Li C., Yang W., Liu J., An X., Zhao Y. (2022). Deep balanced cascade forest: An novel fault diagnosis method for data imbalance. ISA Trans..

[95] Zhang H., Cisse M., Dauphin Y.N., Lopez-Paz D. mixup: Beyond Empirical Risk Minimization. Proceedings of the International Conference on Learning Representations.

[96] Xu K., Feng D., Mi H., Zhu B., Wang D., Zhang L., Cai H., Liu S. (2018). Mixup-based acoustic scene classification using multi-channel convolutional neural network. Proceedings of the Pacific Rim Conference on Multimedia.

[97] Denoyer L., Gallinari P. (2014). Deep sequential neural network. arXiv.

[98] Wu Z., Nagarajan T., Kumar A., Rennie S., Davis L.S., Grauman K., Feris R. BlockDrop: Dynamic Inference Paths in Residual Networks. Proceedings of the IEEE Conference on Computer Vision and Pattern Recognition (CVPR).

[99] Shazeer N., Mirhoseini A., Maziarz K., Davis A., Le Q., Hinton G., Dean J. (2017). Outrageously large neural networks: The sparsely-gated mixture-of-experts layer. arXiv.

[100] Bengio E., Bacon P.L., Pineau J., Precup D. (2015). Conditional computation in neural networks for faster models. arXiv.

